# Biopolymer Composites Filled with Brewer’s Spent Grain for Yeast Immobilization

**DOI:** 10.3390/polym18202576

**Published:** 2026-10-19

**Authors:** Jiayue Liu, Witold Fortuniak, Joanna Bojda, Joanna Zakrzewska, Anna Rygala, Ewa Piorkowska, Dorota Kregiel, Urszula Mizerska

**Affiliations:** 1 Interdisciplinary Doctoral School, Lodz University of Technology, Żeromskiego 116, 90-924 Lodz, Poland; jiayue.liu@dokt.p.lodz.pl; 2 Department of Environmental Biotechnology, Faculty of Biotechnology and Food Sciences, Lodz University of Technology, Wolczanska 171/173, 90-924 Lodz, Poland; 3 Centre of Molecular and Macromolecular Studies Polish Academy of Sciences, Sienkiewicza 112, 90-363 Lodz, Polandjoanna.bojda@cbmm.lodz.pl (J.B.); joanna.zakrzewska@cbmm.lodz.pl (J.Z.)

**Keywords:** biopolymers, poly(3-hydroxybutyrate) (PHB), poly(3-hydroxybutyrate-co-3-hydroxyvalerate) (PHBV), polylactide (PLA), immobilization, *Metschnikowia strains*, natural filler, brewer’s spent grain (BSG), biocomposites

## Abstract

Biopolymer-based composites incorporating natural fillers were developed as carriers for various microbiological applications. Three commercially available biodegradable and bio-based polymers: poly(3-hydroxybutyrate) (PHB), poly(3-hydroxybutyrate-co-3-hydroxyvalerate) (PHBV), and polylactide (PLA)were used as matrices. Brewer’s spent grain (BSG), a by-product of the brewing industry, was employed as a natural filler. A series of biocomposites containing 20, 30, and 40 wt% BSG were fabricated. The structure and thermal properties of the composites were characterized using scanning electron microscopy (SEM) and differential scanning calorimetry (DSC). Surface properties were evaluated by measuring contact angles with two probe liquids, enabling calculation of surface energy and its polar and dispersive components. In turn, microscopic studies were conducted to evaluate the adhesion and colonization of *Metschnikowia andauensis* cells on the prepared carriers. The biocontrol activity of the cell–carrier system was assessed using the agar plate method. The results identified the most promising formulations: PHBV with 40 wt% BSG and PLA with 20–40 wt% BSG, especially with 30 wt% BSG. However, the highest biocontrol efficacy among cells adhered to the biocomposite materials was observed with the last one. This study explored the relationship between the physicochemical properties of BSG-filled biopolymers and their suitability for microbial colonization, highlighting their potential as functional carriers for yeast cells.

## 1. Introduction

Over the past several years, research has been conducted on Metschnikowia yeast strains, demonstrating their potential applications in enology, biocatalysis, agriculture, the food industry, and the cosmetics sector [1,2,3,4]. Among their numerous advantages are their biocontrol properties against phytopathogenic fungi [5,6].

In biotechnological processes involving *Metschnikowia* cells, the concentration of fermentation by-products is influenced by various factors, including the environmental conditions [7,8].

In immobilized cell technology (ICT), high production of metabolites can be achieved at elevated cell densities within a confined volume. This can be accomplished, among other strategies, through cell immobilization. Immobilized yeast cells exhibit enhanced resistance to environmental stress, resulting in higher activity compared to free cells [9,10]. However, this effect is observed only when a sufficiently high cell concentration is maintained [11]. For yeast like the *Metschnikowia* genus, whose metabolism is predominantly aerobic, improved performance is achieved when cells are immobilized on an appropriate carrier rather than entrapped within gel matrices. Consequently, there is a need for solid supports with compositions and/or surface characteristics that promote effective yeast cell adhesion.

Various solid supports, including alginate [12], chitosan [13], biochar [14], carrageenan [15], and synthetic polymers such as methacrylate copolymers [16], have been explored for immobilization purposes. It is also important to consider other biobased and biodegradable polymers, such as poly(3-hydroxybutyrate) (PHB), poly(3-hydroxybutyrate-co-3-hydroxyvalerate) (PHBV), and polylactide (PLA), which are biocompatible, non-toxic, and produced via environmentally friendly processes. As biodegradable materials, they offer an alternative to conventional petrochemical plastics in many sectors of the rapidly growing plastics market [17].

PHB and PHBV belong to the group of polyhydroxyalkanoates that are synthesized as metabolic products of microbial activity. PHB is a crystalline polymer that exhibits effective barrier properties against hydrophobic substances and increased permeability to water vapor. In turn, PHBV contains 3-hydroxyvalerate units, which directly contribute to a reduction in the melting temperature and degree of crystallinity of the polymer [18,19]. Polylactide (PLA) is a biodegradable thermoplastic polymer derived from lactic acid, which is produced through the fermentation of renewable resources. From the perspective of green technologies, the biodegradability of a polymer is highly dependent on the environmental conditions, including temperature, moisture, and microbial activity. Generally, PHB exhibits a broader and more rapid biodegradation profile across various natural environments compared to PLA [20,21]. The biodegradability of polymeric materials can be enhanced by the incorporation of natural fillers or bio-based additives, which accelerate and facilitate degradation processes [22].

Numerous studies on PLA and PHB reinforced with natural additives have primarily focused on their influence on the crystallization, structure and properties of the resulting composite [23,24,25,26,27,28,29,30,31,32]; a variety of natural fillers have been investigated, including lignin and cellulose [28,29], rice husks [25], and hemp fibers, as well as agave fibers and wheat flour [30,31,32].

In the present study, brewer’s spent grain (BSG), a by-product of the brewing industry, was employed as a filler. According to available statistics, breweries in Europe generate approximately 3.4 million tons of BSG annually [33]. BSG contains about 70 wt.% dietary fiber and can therefore be considered a lignocellulosic material. In addition, it comprises approximately 25 wt.% moisture and 20 wt.% protein, as well as phenolic compounds and melanoidins [34]. As a result, BSG is widely used as a low-cost, approximately €35 per ton [35] feed for dairy cattle, which does not sufficiently alleviate the challenges of large-scale, continuous production. However, due to its moisture content and susceptibility to microbial growth, it requires rapid utilization or disposal.

Previous studies have investigated the use of BSG as a filler in various polymer matrices, including poly(butylene succinate) and poly(hydroxybutyrate-co-hydroxy-hexanoate) [36], poly(ε-caprolactone) [37], polylactide and polypropylene [38], as well as polyurethane [39].

In the present study, a series of biopolymer-based composite carriers were fabricated by incorporating BSG at 20, 30, and 40 wt.% content. The samples were subsequently immersed in a culture medium inoculated with *Metschnikowia andauensis* (*M. andauensis*) yeast cells. After cultivation, carriers were subjected to extensive physicochemical and microbiological analyses, which included: contact angle, water absorption tests, thermal analysis, microscopic techniques, and the number of adhered yeast cells and their biocontrol activity against phytopathogen *Verticillium* sp. [40,41]. For each polymer matrix, the formulation exhibiting the most favorable performance was selected.

## 2. Materials and Methods

### 2.1. Materials

The study utilized commercially available polymers from manufacturer TianAn-ENMAT (Ningbo City, China): poly(3-hydroxybutyrate) Enmat Y3000P (PHB) and poly(3-hydroxybutyrate-co-3-hydroxyvalerate) Enmat Y1000P (PHBV). Both polymers contain 1% boron nitride and 0.5% antioxidant KY1010. A polylactide with a D-lactide content of about 1% determined using polarimetry, purchased from Nature Works (Blair, NE, USA), NW4032D, was used. The average molar masses—number (Mn), weight (Mw), and z-average (Mz)—were 76 kg/mol, 109 kg/mol, and 122 kg/mol, respectively, measured for PLA by size-exclusion chromatography (SEC) with a multi-angle laser light-scattering (MALLS) detector in CH_2_Cl_2_.

Brewer’s spent grain (BSG), a by-product of beer production, was obtained from DRINK ID Company located in Piotrkow Trybunalski, Poland.

The *M. andauensis* D6 and *Verticillium tenerum* (*V. tenerum*) strains used in this study were obtained from the LOCK 105 Culture Collection, Lodz, Poland [41].

### 2.2. Characterization of Brewer’s Spent Grain (BSG)

The BSG was characterized with scanning electron microscopy (SEM) using a JSM-6010LA microscope (JEOL, Tokyo, Japan) at an accelerating voltage of 15 kV.

To determine the moisture content and thermal stability of BSG, thermogravimetric analysis (TGA) was performed using a TGA 5500 from TA Instruments (New Castle, DE, USA) under a nitrogen flow of 60 mL/min during heating from 25 to 800 °C at a rate of 20 °C/min.

### 2.3. Preparation of Carriers

#### 2.3.1. Pre-Treatment of Brewer’s Spent Grain (BSG)

BSG, with an initial moisture content of approximately 80%, was dried on trays at 40–50 °C for four days, with stirring every few hours to ensure uniform drying. The moisture content in the dried BSG was approximately 12 wt.%, as determined using a MA 50R moisture analyzer (Radwag, Radom, Poland). The dried grains were then ground in an electric mill (Gallet HS703, ETA a.s., Prague, Czechia) and sieved to obtain particles with sizes up to 3 mm (3 mm laboratory sieve, Labindex, Warsaw, Poland).

A portion of BSG was mixed with tap water at a weight ratio of 1:5 and heated in a water bath at 90 °C for 1 h. The mixture was then left to cool gradually to room temperature in the switched-off water bath for 12 h. Following aqueous extraction, the BSG biomass was dried under the previously described conditions to reduce its moisture content to below 3 wt.%.

#### 2.3.2. Carrier Preparation

Biopolymer composites containing 20, 30, and 40 wt.% BSG were prepared by melt-mixing the biopolymers with milled BSG in a co-rotating, intermeshing twin-screw extruder (Zamak, Skawina, Poland) at barrel temperatures ranging from 160 to 185 °C and a screw speed of 60 rpm. Neat biopolymers were processed under the same conditions. Prior to processing, the biopolymers were dried under reduced pressure at 80–100 °C for 4 h. The biocomposites are designated, for example, as PLA20, where the number following the polymer abbreviation indicates the BSG content (wt.%).

**Figure 1:**
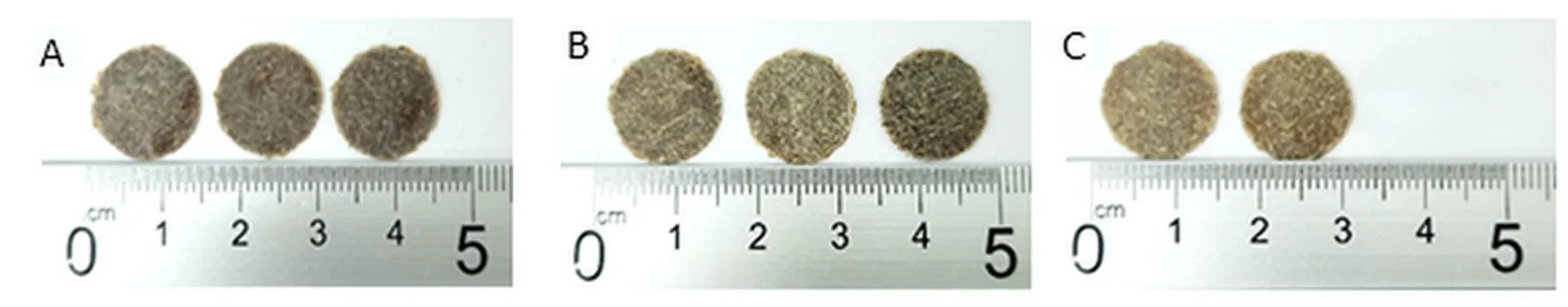
Photographs of BSG-containing carriers: (**A**) PLA-based, (**B**) PHBV-based, and (**C**) PHB -based. BSG content increases from left to right: 20 wt.%, 30 wt.% and 40 wt.%.

The prepared materials were compression molded into films approximately 0.5 mm thick at 180 °C under a pressure of 50 MPa, followed by quenching between metal plates maintained at room temperature. Subsequently, disks with a diameter of 12 mm, shown in Figure 1, were cut out from the films and used as carriers for the microbiological tests.

### 2.4. Carrier Characterization

#### 2.4.1. Scanning Electron Microscopy

The carrier samples were analyzed by scanning electron microscopy (SEM) using JEOL JSM-6010LA and JSM-5500LV microscopes (Tokyo, Japan) operated at accelerating voltages of 15 kV and 10 kV, respectively. To examine their internal structure, the carrier films were cryo-fractured. In addition, the surfaces of dried carriers with yeast cells, after cultivation, were placed on a copper tape and examined by SEM. Prior to examination, all samples were sputter-coated with gold.

#### 2.4.2. Light Microscopy

The carrier samples were examined using a DSX2000 digital microscope (Evident, Tokyo, Japan) equipped with a telecentric optical system and a high-resolution camera, enabling imaging over a wide magnification range with optical zoom.

#### 2.4.3. Differential Scanning Calorimetry

The carrier samples were studied with differential scanning calorimetry (DSC) using a Discovery DSC 2500 calorimeter (TA Instruments, New Castle, DE, USA). The specimens were heated to 200 °C and held at this temperature for 3 min, then cooled and heated again, all at a rate of 10 °C/min under a nitrogen flow.

#### 2.4.4. Thermogravimetry

Thermogravimetric analysis (TGA) of the carrier samples was performed using a TGA 5500 analyzer (TA Instruments, New Castle, DE, USA). The measurements were carried out under a nitrogen flow of 60 mL/min during heating from 25 to 800 °C at a rate of 20 °C/min. The measurements were repeated two or three times for every sample type.

#### 2.4.5. Contact Angle Measurements

Contact angle measurements were performed using a Phoenix-300T analyzer (Surface Electro Optics, Suwon City, Kyunggido, Republic of Korea) with Surfaceware v7 software. Droplets of 5 μL of test liquids, water and diiodomethane, were deposited onto the surfaces of the carriers, and their images were recorded and analyzed. The surface free energy and its dispersive and polar components were determined using the Owens–Wendt approach. The calculations were performed using Drop v3 software.

#### 2.4.6. Water Absorption Tests

Water absorption was evaluated using carrier disks with a diameter of 12 mm cut from 0.5 mm thick films. Prior to testing, the carrier disks were dried in an oven at 50 °C for 24 h. They were then immersed in distilled water at room temperature. After 4 and 14 days of immersion, the carrier disks were removed from the water, gently blotted dry with absorbent paper, and weighed immediately. The water uptake was calculated from the increase in sample weight. Three carrier disks of each material were tested, and the average value was calculated.

### 2.5. Microbiological Analysis

#### 2.5.1. Yeast Cultivation on Media

In this study, the *M. andauensis* D6 strain, exhibiting biocontrol activity against *Verticillium* sp., was used [40,41]. Yeast cells were cultivated in liquid wort broth (Merck, Darmstadt, Germany) in the presence of polymeric carriers. Cultivation was performed in sterile Petri dishes containing 20 mL of medium, up to five carrier samples, and 1% (*v*/*v*) yeast inoculum with a density of 1–2 on the McFarland scale [°McF] (DEN-1 densitometer, Merck, Darmstadt, Germany). The cultures were incubated at 25 °C with agitation at 75 rpm (Unimax 1010, Heidolph, Germany) for 6 days [10].

#### 2.5.2. Determination of Number of Adhered Yeast Cells

After cultivation, each carrier from the experimental variants was mechanically fragmented and transferred into a sterile glass tube containing 5 mL of Ringer’s solution and two drops of Tween 80 (Sigma Aldrich, St. Louis, MO, USA). Then, the samples were vortexed for 15 min to detach adhered cells. The resulting cell suspensions were analyzed microscopically using a light microscope (Olympus CX41, Tokyo, Japan) and a Thoma counting chamber (Marienfeld-Superior, Lauda-Königshofen, Germany) to determine cell numbers. The results are reported as the mean number of cells per carrier from three independent replicates, along with the corresponding standard deviation. In addition, the surfaces of carriers after yeast cultivation and drying were examined by SEM, as described above.

#### 2.5.3. Biocontrol Activity of Adhered Cells

The in vitro assessment of the antifungal activity of the *M. andauensis* D6 strain against the phytopathogenic fungus *Verticillium* was conducted using the modified agar cross method [42]. The *M. andauensis* was cultured in wort broth (Merck, Darmstadt, Germany) for 6 days at 25 °C on a rotary shaker (Unimax 1010, Heidolph, Schwabach, Germany) at 140 rpm. The *Verticillium* strain was cultivated on PDA agar (Merck, Darmstadt, Germany) for 5 days at 25 °C. A 100 µL sample of a freshly prepared phytopathogen cell suspension in Ringer solution, measured densitometrically in MacFarland units (2 °McF), was inoculated onto PDA plates. Carriers with adhered yeast cells were then transferred onto these agar plates. The samples were incubated at 25 °C for 5 days. The diameters of pathogen growth inhibition zones around the carriers were measured manually. The results were expressed as the Q factor, calculated as the ratio of the inhibition zone diameter to the carrier diameter. Results are the averages of three independent replicates with standard deviation.

## 3. Results

### 3.1. Structure

**Figure 2:**
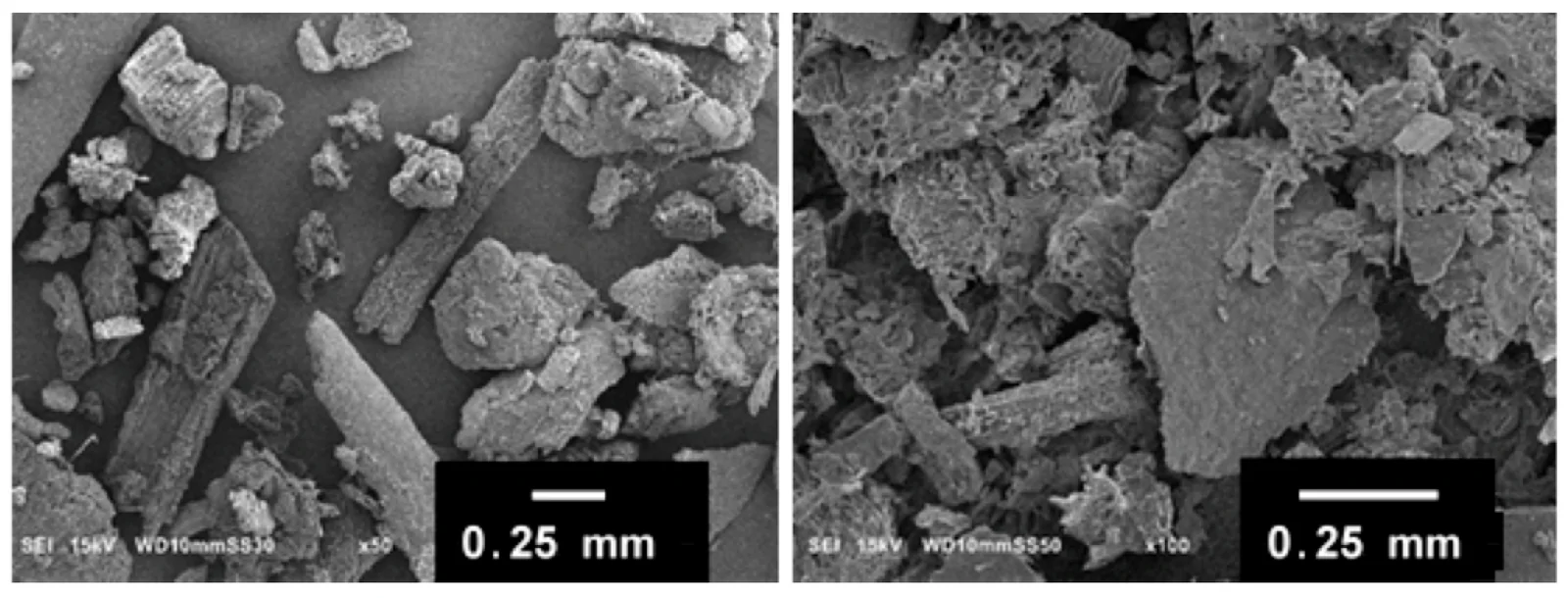
SEM micrographs of milled grains of BSG.

The SEM analysis confirmed that the irregular and differently shaped BSG particles, including rod-like ones, were up to 3 mm in size, as shown in Figure 2. TGA thermogram of BSG is presented in Figure S1 in the Supplementary Materials (SM). A weight loss of about 3% occurred at the initial stage of heating, below 100 °C, and was associated with the evaporation of residual moisture. Fast weight loss began above 200 °C and lasted to about 450 °C, with two maxima of the weight-loss rate at 298 and 355 °C. At 200 °C, weight loss reached only 4%. The thermal stability of BSG up to 200 °C made it possible to mix BSG with the molten biopolymers.

**Figure 3:**
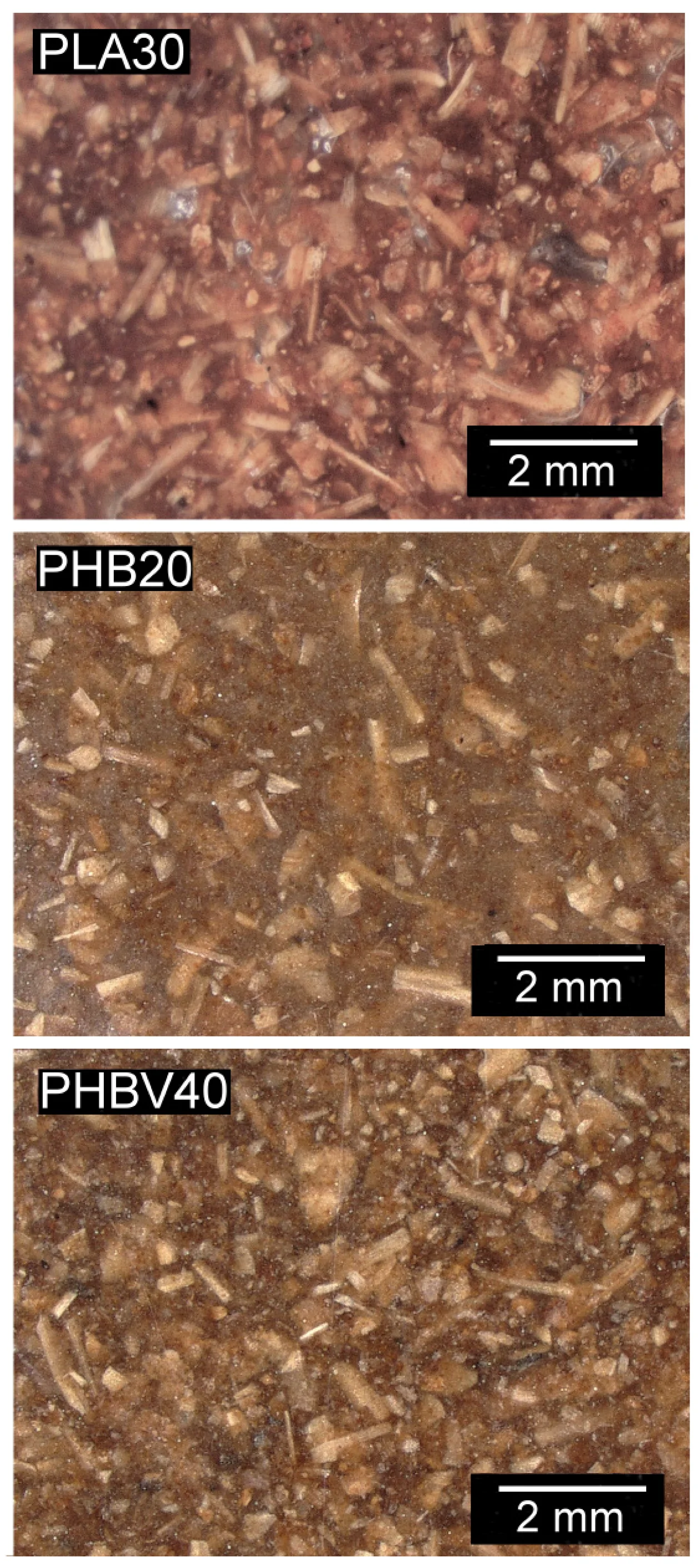
Light microscopy images of biocomposite carriers: PLA30, PHB20 and PHBV40 in reflected light.

Figure 3 presents exemplary light micrographs of selected biocomposites. The images reveal the presence of BSG particles, with different shapes, whether irregular or elongated rod-like, and fine particle fractions. The micrographs indicate good dispersion of the filler within the polymer matrices, which is further supported by SEM analysis.

**Figure 4:**
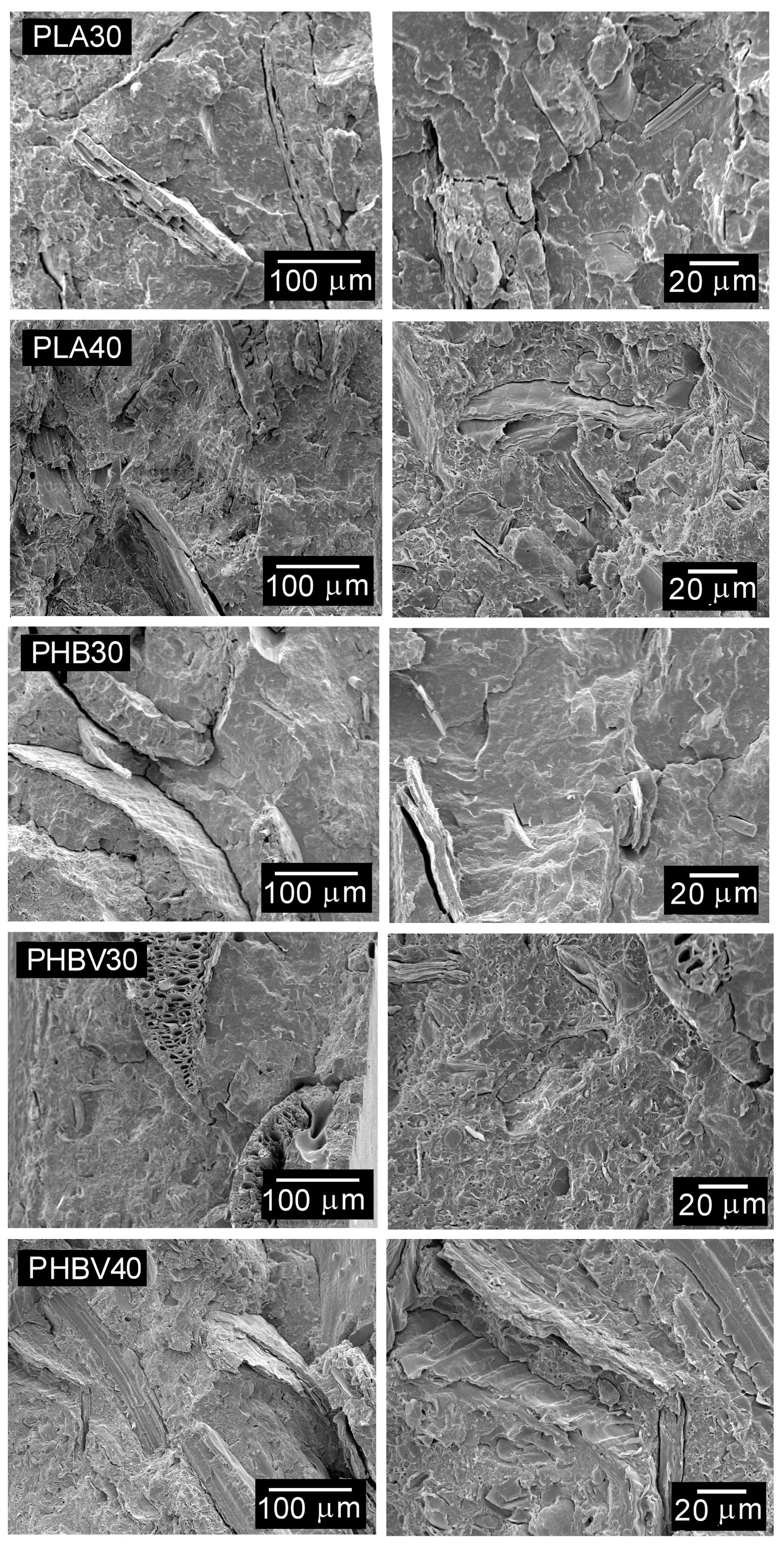
Scanning electron micrographs of cryo-fracture surfaces of biocomposite carriers: PLA30, PLA40, PHB30, PHBV30 and PHBV40.

SEM micrographs of the cryo-fracture surfaces of the biocomposite carriers with 30–40 wt.% BSG are shown in Figure 4, whereas those of corresponding neat biopolymer carriers are presented in Figure S2 in SM for comparison. The neat polymers exhibit brittle fracture surfaces, which are less developed for the amorphous PLA and more pronounced for the semi-crystalline PHB and PHBV. The micrographs of the biocomposite carriers reveal BSG particles of various sizes, as well as voids left by particles that were detached from the polymer matrices during fracture. The images indicate that the BSG particles were well dispersed within the polymer matrices, with no evidence of filler agglomeration. The micrographs show both large BSG particles and numerous fine particles. The latter are clearly visible in the micrographs presented in Figure S3 in SM. The fine particles were likely generated either during BSG milling or during melt mixing with the biopolymers.

### 3.2. Thermal Properties and Crystallinity

**Figure 5:**
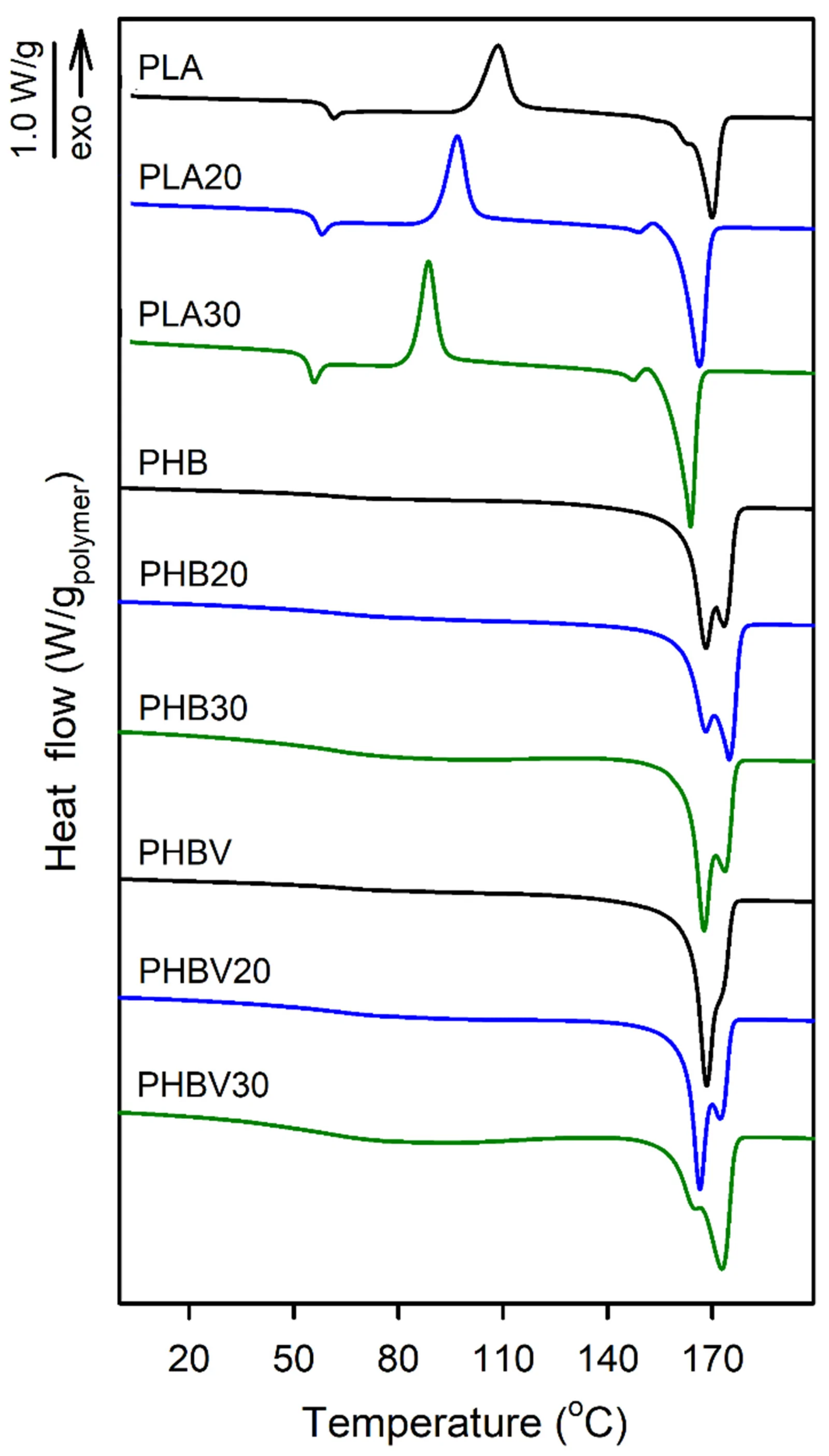
DSC first-heating thermograms of PLA-, PHB- and PHBV-based materials recorded at 10 °C/min.

DSC first-heating thermograms of the selected carrier samples are presented in Figure 5. The thermogram of each PLA-based material exhibits a glass transition, a cold-crystallization exotherm, and a melting endotherm. The glass transition temperature (T_g_) of neat PLA was 58.5 °C. The T_g_ of PLA20 was 55 °C, whereas that of PLA30 and PLA40 was 53.5 °C. This decrease was most likely caused by plasticization by compounds released from BSG during melt blending. The cold-crystallization peak temperature decreased from 108 °C for neat PLA to 97 °C for PLA20 and to 89–92 °C for the biocomposites containing 30–40 wt.% BSG. The melting peak temperature (T_m_) was the highest for neat PLA (170 °C) and lower for the biocomposites (164–166 °C). A small pre-melting exotherm evidenced the recrystallization of the disordered α′ phase into the ordered α phase [43]. The melting enthalpy (ΔHm) of the biocomposites, 46–53 J/g_PLA_, exceeded the cold-crystallization enthalpy, 39–43 J/g_PLA_, evidencing the crystallinity of these materials prior to heating in DSC. The degree of crystallinity, calculated assuming a heat of fusion of 106 J/g for 100% crystalline PLA [44], although low, increased with increasing filler content to 3, 9, and 13% at 20, 30, and 40 wt.% BSG, respectively. The crystallization of the PLA biocomposite carriers upon cooling during film preparation, undoubtedly caused by nucleation on BSG, was confirmed by small crystallization exotherms observed on the DSC cooling thermograms shown in Figure S3 in SM. In turn, the melting and cold-crystallization enthalpies of 37 J/g were equal for the neat PLA carrier, indicating that it was amorphous prior to DSC heating.

The thermograms of the PHB-based materials exhibited pronounced double melting endotherms, with peaks at 168 and 173–174 °C, attributed to melting followed by recrystallization upon heating [45]. The ΔH_m_ of 90–92 J/g_PHB_ corresponded to a crystallinity degree of approximately 60 wt.%, assuming a heat of fusion of 146 J/g for 100% crystalline PHB [46]. The PHBV-based materials behaved similarly. A single melting peak at 168 °C with a high-temperature shoulder, observed for the neat PHBV, changed into a double melting endotherm for the biocomposites, with peaks at temperatures of 172 °C and 163–166 °C, the temperature decreasing with increasing BSG content. The crystallinity degree calculated from ΔH_m_ was similar to that of the PHB-based materials—approximately 60 wt.%.

The completion of crystallization during cooling in the PHB- and PHBV-based materials was confirmed by the DSC cooling thermograms presented in Figure S4 in SM, showing crystallization exotherms with peak temperatures of 114–119 °C and 111–119 °C, respectively. These temperatures decreased with increasing BSG content because the volume occupied by BSG was inaccessible for the nucleation of polycrystalline aggregates and acted as an obstacle to their growth. The T_g_ values of PHB and PHBV containing 2% of 3-hydroxy-valerate units are close to 5 and 1.5 °C, respectively [47,48]. However, the glass transition was not clearly recognizable in the heating thermograms of the PHB- and PHBV-based materials because of their relatively high crystallinity. In turn, weak signals related to the relaxation of the rigid amorphous phase appeared in the temperature range of 50–70 °C [49]. The endothermic effect in this temperature range could have been enhanced by the evaporation of residual water from BSG, as observed in the TGA thermograms and discussed below. This effect was not observed in the second-heating thermograms recorded immediately after cooling.

The TGA thermograms of the neat biopolymer and biocomposite carriers containing the highest BSG contents are shown in Figure S1 in SM. At the beginning of heating, the biocomposites exhibited only small weight losses of 1.8–2.8%, most likely due to the evaporation of residual moisture present in the BSG. The weights of PHB30, PHBV40, and PLA40 started to decrease markedly at approximately 225 °C, whereas those of neat PHB and PHBV started to decrease significantly only above 250 °C and that of neat PLA above 300 °C. The earlier weight loss of the biocomposites was attributed to the weight loss of BSG increasing above 200 °C. A 10% weight loss was reached at about 280 °C for PHBV40 and PHB30, compared with about 290 °C for the respective neat biopolymers. However, the temperatures corresponding to 50% weight loss were very similar 303 °C for the biocomposites and 305 °C for the neat polymers. Likewise, the temperatures of the fastest weight loss differed only slightly, being 298–300 °C for the biocomposites and 303–304 °C for the neat biopolymers, with the higher values observed for the PHBV-based materials. The stronger effect of BSG on the thermal stability of PLA40 was reflected in the temperature corresponding to 10% weight loss, which decreased from 348 °C for neat PLA to 296 °C for PLA40. The temperatures corresponding to 50% weight loss and to the fastest weight loss were also reduced, by about 12 °C, from 377–378 °C for neat PLA to 365–366 °C for PLA40. At 450 °C, the remaining weight of the biocomposites was mainly associated with the BSG residue. Besides the weight loss of BSG in the biocomposites, the products released during BSG decomposition could trigger the earlier degradation of the PLA matrix, as reported for PLA filled with other cellulose-containing fillers [50]. In addition, residual moisture and hydroxyl groups present in BSG could facilitate the hydrolytic degradation of PLA. Similar effects have also been reported for PHB and PHBV composites containing cellulose-based fillers [51,52]. Nevertheless, all the investigated materials were thermally stable within the temperature range of their processing, not exceeding 185 °C.

### 3.3. Surface Properties

Contact angle values of the carriers are listed in Table 1. The changes are of the order of several degrees and are within the limits of experimental data scatter. They might be caused by the presence of hydrophilic BSG particles on the surface, which could enhance hydrophilicity but also increase surface roughness causing the opposite effect.

**Figure 6:**
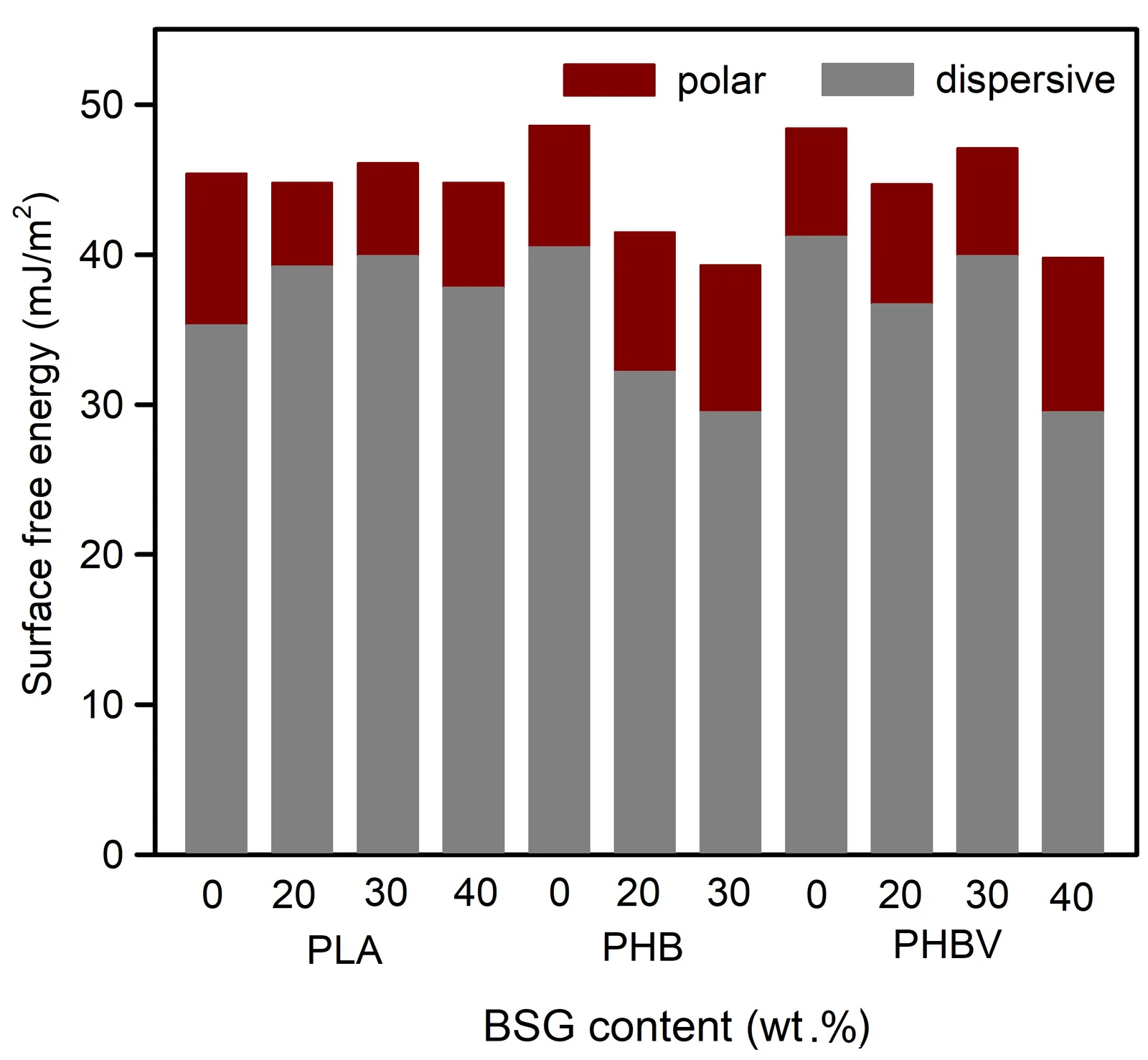
Surface free energy of PLA-, PHB- and PHBV-based carriers.

Based on the contact angle values measured for two probe liquids, the surface free energy was determined using the Owens–Wendt method, and the results are presented in Figure 6. For all tested carriers, the dispersive component of the surface free energy dominated over the polar component. The dispersive component reflects nonpolar van der Waals interactions, while the polar component represents specific interactions such as hydrogen bonds and dipole–dipole forces. The ratio of these two components determines surface wettability and strongly influences microorganism–surface interactions, including cell adhesion and colonization.

In PLA-based systems, the incorporation of BSG caused only minor changes in the surface free energy values. A slight decrease in the polar component was observed, accompanied by a corresponding increase in the dispersive contribution. Consequently, the total surface free energy of the PLA biocomposites remained relatively stable, in the range of 44.8–46.1 mJ/m^2^, suggesting that the addition of BSG did not significantly alter the balance of intermolecular interactions at the surface. This may be related to the relatively good compatibility between PLA and the filler, leading to limited exposure of highly polar BSG filler components at the surface.

More pronounced changes were observed for the PHB- and PHBV-based materials. The presence of BSG in the PHB-based biocomposites resulted in a measurable decrease in the total surface free energy from 48.6 to 39.3 mJ/m^2^. This effect was associated with changes in the relative contribution of the surface energy components—specifically, an increase in the polar component, from 8.1 to 10.2 mJ/m^2^, coupled with a decrease in the dispersive component, from 40.5 to 29.1 mJ/m^2^. Similarly, for PHBV-based biocomposites, a decrease in total surface free energy from 48.4 to 39.7 mJ/m^2^ was observed, coupled with equivalent changes in the dispersion and polar components as for PHB. This different behavior can be attributed to differences in polymer–filler interfacial interactions, leading to exposure of BSG at the carrier surfaces.

Overall, the results indicate that although BSG addition modifies the surface-energy characteristics of biopolymer composites, the extent and direction of these changes depend on the nature of the polymer matrix. These findings are particularly relevant in the context of microbial adhesion, as both the magnitude of surface free energy and the balance between polar and dispersive interactions are known to influence interactions at the cell–surface interface.

### 3.4. Water Absorption

**Figure 7:**
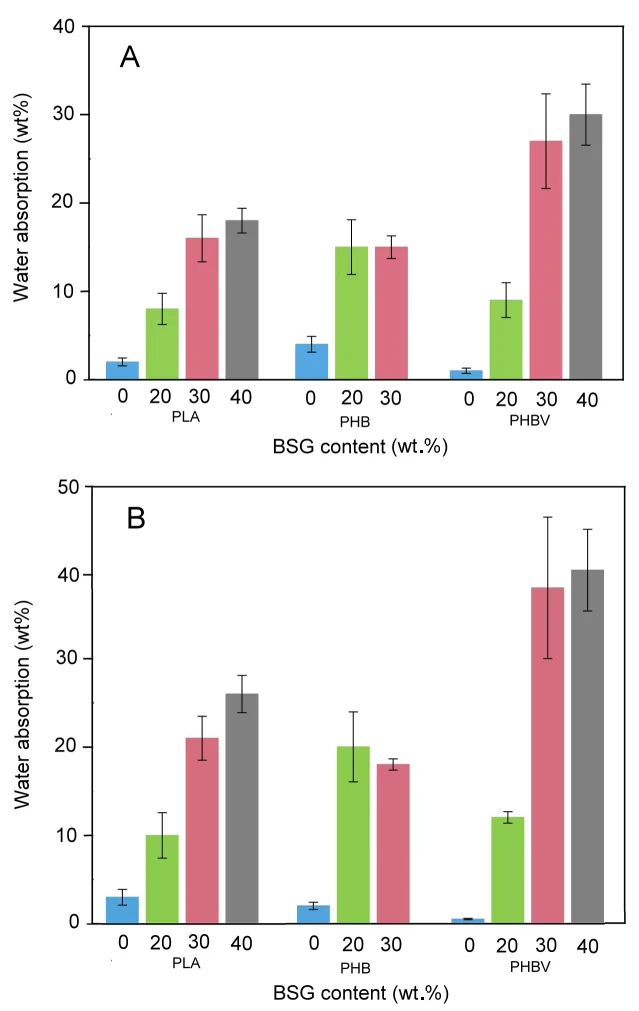
Water absorption of PLA-, PHB-, and PHBV-based carriers after immersion in water for 4 days (**A**) and 14 days (**B**).

Water absorption, expressed as wt.% relative to the dry mass after immersion in water for 4 and 14 days, is presented in Figure 7. Long-term immersion caused only minor changes, indicating that equilibrium water absorption was achieved after 14 days. Neat biopolymer carriers absorbed only a few percent of water. However, the biocomposites with BSG exhibited increased water absorption, primarily due to the hygroscopicity of the filler particles. The largest values of water absorption were exhibited by PHBV-based materials.

### 3.5. Growth of Yeast Cells on Carriers

**Figure 8:**
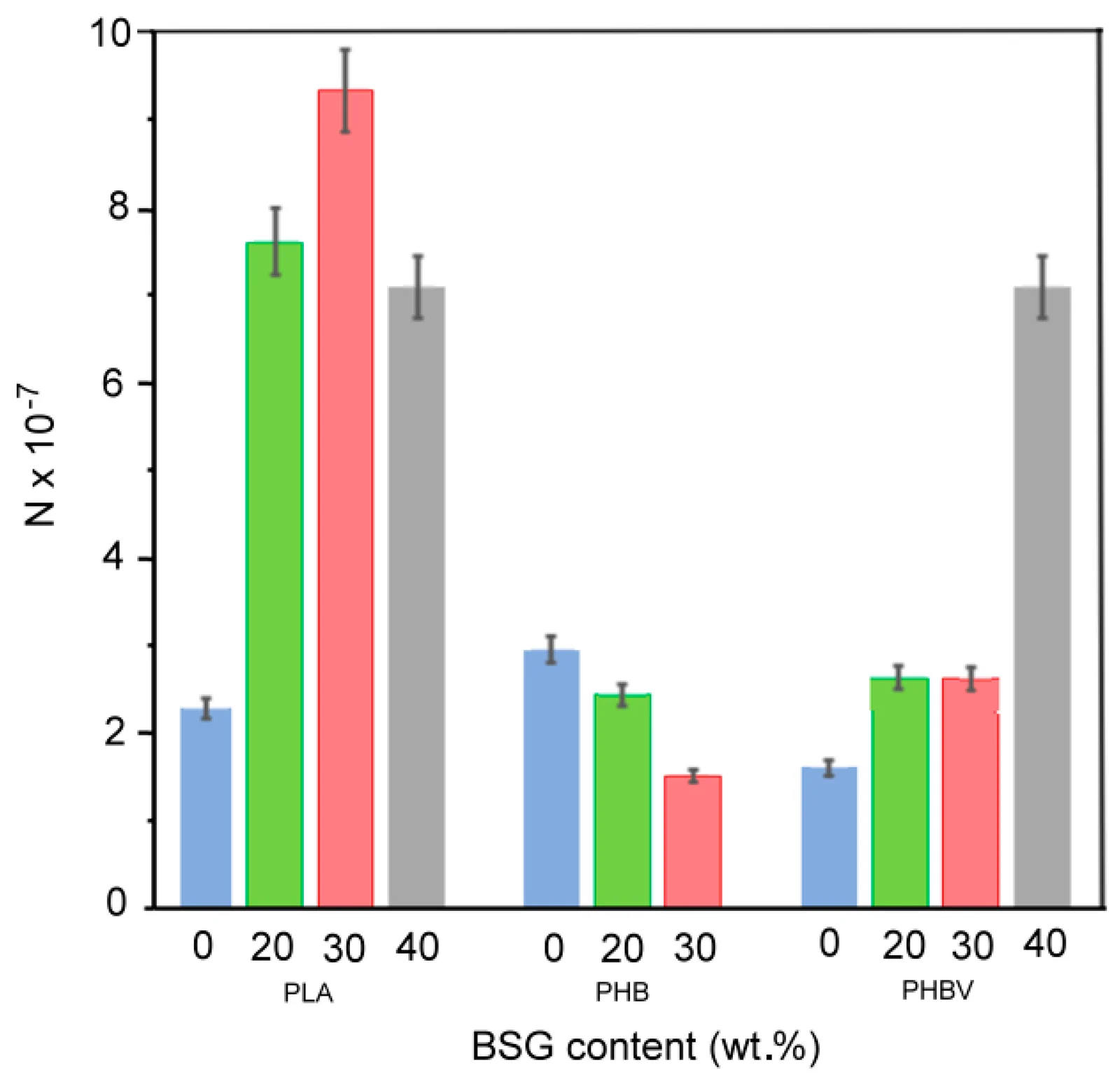
Yeast multiplication on PLA-, PHB- and PHBV-based carriers after 6 days of cultivation (*p* < 0.05). N denotes the number of cells.

To identify the most suitable carriers among the prepared biocomposites, *M. andauensis* D6 strain was cultured on the materials, and the number of adhered yeast cells was subsequently quantified. The results, expressed as the number of counted cells obtained using microscopic analysis, are presented in Figure 8 and in Table S1 in SM.

**Figure 9:**
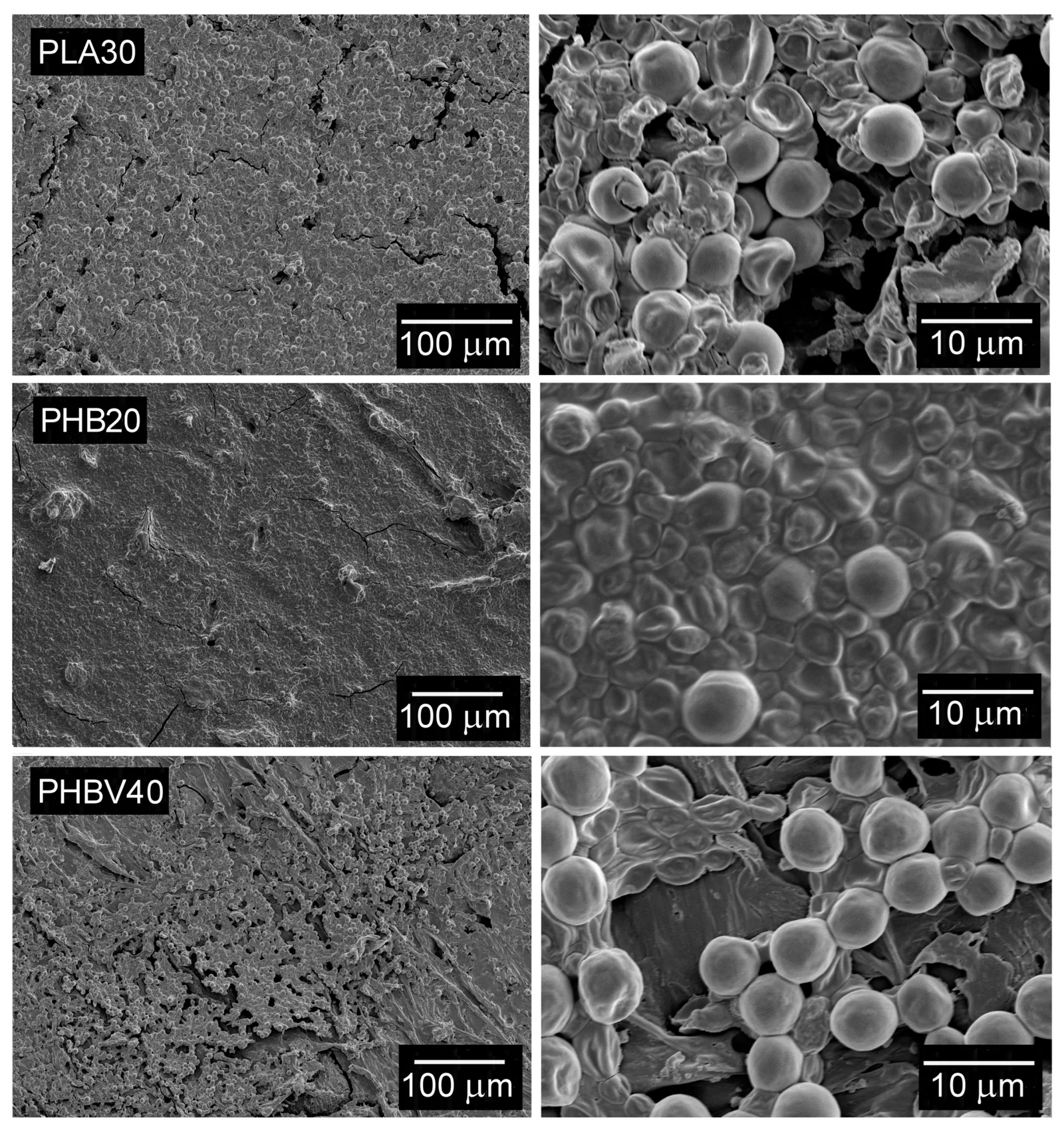
SEM micrographs of biocomposite carriers, PLA30, PHB20, and PHBV40 after 6 days of *M. andauensis* D6 cultivation.

Preliminary experiments were conducted to assess yeast proliferation over a short incubation period of 24 h. Subsequently, extended cultivation studies were performed under identical conditions for 6 days. Microscopic observations revealed that, after prolonged incubation, the majority of the carrier surface was covered with microcolonies of *M. andauensis* D6, as confirmed by the SEM images shown in Figure 9.

This behavior is indicative of favorable growth conditions provided by the carrier surface. The formation of microcolonies, arranged as layered structures on the surface of abiotic materials, corresponds to the development of biofilms. Biofilm formation is a common microbial strategy, with cells in such structures exhibiting enhanced resistance to environmental stress and improved survival compared to planktonic counterparts [53].

The biocomposite carriers supported high cell densities, reaching up to 7.5 × 10^8^ cells per carrier, demonstrating that the developed material provides a highly conducive environment for the growth and colonization of *M. andauensis* cells. The best results were obtained for the PLA-based biocomposite carriers containing 20–40 wt.% BSG and for PHBV40. Among the PLA-based carriers, the highest cell density was observed on PLA30. Of the two PHB-based carriers, the higher cell density was observed for PHB30; however, it was lower than that obtained for PHBV40 and all PLA-based carriers containing BSG.

### 3.6. Biocontrol Activity of Adhered Yeast Cells

**Figure 10:**
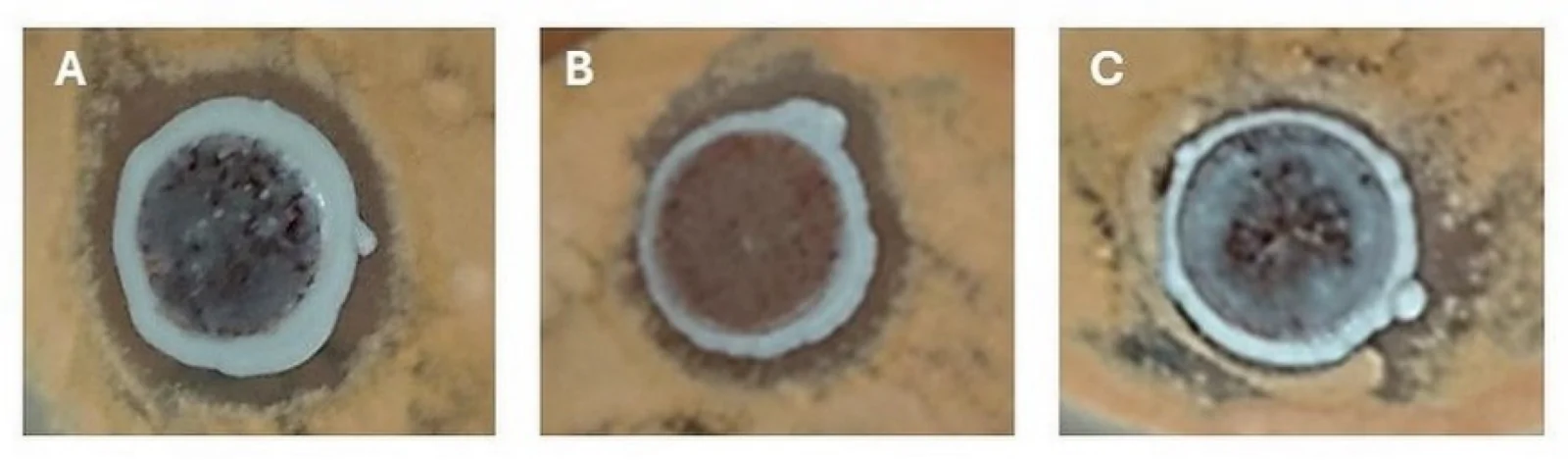
Antifungal activity of the *M. andauensis* D6 strain adhered to the surface of the carriers: (**A**) PLA30; (**B**) PHB20; (**C**) PHBV40 in the plate test against *Verticillium* sp.

Our previous studies demonstrated that free (planktonic) cells of the *M. andauensis D6* strain exhibit significant biocontrol activity against phytopathogen *Verticillium* sp. [40,41]. Based on these findings, the present study aimed to evaluate whether this antagonistic activity is retained after immobilization of the yeast cells on the biocomposite carriers. To assess this, biocontrol assays were performed using a cross method on Petri dishes containing PDA medium inoculated with the target phytopathogen. The most efficient BSG-containing carriers based on PLA, PHB and PHBV were tested. The inhibitory effect of immobilized yeast cells on pathogen growth was subsequently evaluated. The results of these experiments are presented in Figure 10.

For the biocontrol experiments, three carrier variants exhibiting the highest yeast cell densities were selected (Figure 10). The antifungal activity of the *M. andauensis* strain immobilized on these carriers varied depending on the composite formulation. Among the tested materials, the PLA-based composite containing 30 wt.% BSG demonstrated the most pronounced biocontrol effect, as evidenced by the largest inhibition zones and the highest calculated inhibition coefficient (Q) equal 1.84 ± 0.31 (Table 2).

*Verticillium* sp. are soil-borne phytopathogens that exhibit pronounced activity within the rhizosphere and soil environment, where they persist via long-lived microsclerotia and infect host plants through root systems [54]. Consequently, biocomposite carriers containing immobilized *M. andauensis* cells with antifungal activity represent a promising strategy for soil application, serving both as biocontrol agents against pathogenic fungi and as biofertilizers that may enhance soil health and plant growth.

PHB has gained attention as a sustainable material, particularly in agricultural applications. However, while this polymer and its degradation products are generally considered non-toxic, their presence in soil can significantly influence soil quality and microbial dynamics, with important consequences for plant growth. Studies by Brtnicky et al. [55] demonstrated that the addition of this polymer to soil creates a highly active microbial environment that enhances biodegradation and nutrient cycling but also disrupts plant–microbe interactions by increasing microbial competition for essential nutrients. Microbial inoculation can partially improve soil microbiota composition and biochemical processes; however, it does not fully restore plant growth. This may also apply to PHBV.

In contrast, PLA is a compostable polymer, and its biodegradation in soil at ambient temperature may take several years because of the slow hydrolysis of its ester bonds. However, plant-fiber reinforcement increases the permeability of PLA to water, thereby promoting hydrolytic degradation of the polymer matrix. As a consequence, the material becomes more susceptible to subsequent microbial biodegradation in soil or compost environments [56]. The incorporation of BSG is expected to facilitate water penetration into the biocomposite and promote the hydrolytic degradation of the PLA matrix, which may subsequently enhance biodegradation under soil or compost conditions.

The enhanced colonization of the PLA-based carriers may have resulted from several contributing factors. Surface free energy may influence interactions at the cell–surface interface. Among the PLA-based carriers, PLA30 exhibited both the highest surface free energy and the greatest yeast colonization, although the differences in surface free energy between these carriers were minor. A similar trend was observed for the PHB-based carriers. However, among the PHBV-based carriers, the sample containing 40 wt.% BSG supported the greatest yeast colonization despite having the lowest surface free energy. The addition of BSG reduced the glass transition temperature of PLA by 3.5–5 °C, indicating partial plasticization of the amorphous phase by low-molecular-weight compounds originating from BSG. Since migration of plasticizers and other low-molecular-weight species toward the surface of amorphous or weakly crystalline polymers has been widely reported, these compounds may also have modified the surface properties of the PLA carriers, creating a more favorable environment for yeast attachment and growth. In addition, crystallinity of the polymer matrix in PLA30 was lower than that of PLA40. For PHBV biocomposites, the improved colonization observed for PHBV40 may instead be associated with the greater content and exposure of BSG domains at the surface enhancing water absorption. However, the absence of a clear correlation between surface energy or water absorption and yeast colonization for all materials indicates that no single parameter governed cell attachment and that the observed behavior likely resulted from the combined effects of surface chemistry and morphology.

When carriers with the same BSG content of 20 or 30 wt.% were compared, PLA-based carriers showed the greatest yeast colonization, followed by PHBV-based then PHB-based carriers, although the differences between the latter two were small. Colonization of PLA40 and PHBV40 was similar but somewhat lower than that of PLA20 and PLA30. It is worth emphasizing that differences in polymer surface properties and interactions at the polymer–BSG interface can lead to variations in the intensity of yeast proliferation on the tested carriers. Surface free energy, wettability, roughness, and the degree of BSG particle exposure on the composite surface are parameters known to influence microbial adhesion and growth [57]. Our research suggests that yeast proliferation is determined not only by the properties of the polymer matrix itself but also by the BSG content and the availability of nutrients derived from the BSG. These factors may partially mask the expected relationship stemming from the polymer’s properties. Yeast adhesion to abiotic surfaces is controlled by a combination of physicochemical factors, and enhanced adhesion generally promotes subsequent colonization, which may ultimately translate into higher yeast proliferation on specific supports.

## 4. Conclusions

This study demonstrated the feasibility of producing biopolymer-based biocomposites containing brewer’s spent grain (BSG) as a natural cellulose filler for potential microbiological applications. Biodegradable polymer matrices—PHB, PHBV, and PLA—were combined with varying amounts of BSG to obtain a series of functional biocomposites. Characterization of these materials showed that the incorporation of BSG influenced their structure, thermal, and surface properties, and determines their suitability as supports for microbial cells. However, the relationships between physicochemical properties, such as surface free energy, wettability and water absorption, and the ability of *M. andauensis* cells to adhere, colonize the carriers, and exhibit biocontrol activity is not straightforward. Surface free energy or water absorption alone cannot account for the observed differences in yeast colonization of the carriers. Among the tested PLA-, PHB-, and PHBV-based biocomposites, PLA30, PHB20, and PHBV40 proved to be the most promising representatives of their respective groups. However, the best performance was achieved by all PLA-based biocomposite carriers and PHBV40.

It should be noted that, after the adhesion experiments with *M. andauensis*, the biocomposites with adhered yeast cells were further used to evaluate their biocontrol efficacy against a phytopathogen. PLA30 exhibited the highest biocontrol efficiency against *Verticillium* sp., indicating its strong potential for practical applications.

These findings highlight the importance of tailoring biocomposite composition to optimize both material properties and biological performance. The study confirms that BSG-filled biopolymer biocomposites can serve as effective and sustainable carriers for beneficial microorganisms, supporting their potential application in agricultural biocontrol strategies.

The most promising of all was the PLA30 biocomposite. Further studies should evaluate its functionality under real soil conditions, especially, its effectiveness in controlling soil-borne phytopathogens that threaten crop health in agriculture should be assessed. Expanding the scope to include other beneficial microorganisms and optimizing the carrier composition for large-scale applications may contribute to the development of sustainable and environmentally friendly agricultural technologies.

## Figures and Tables

**Table 1:** Contact angle values of PLA-, PHB- and PHBV-based carriers.

Sample Code	Contact Angle (Deg)	
	Water	Diiodomethane
PLA	68.2 ± 2.7	35.0 ± 4.9
PLA20	75.7 ± 3.1	31.0 ± 6.0
PLA30	73.8 ± 3.0	28.4 ± 2.7
PLA40	73.1 ± 2.7	32.6 ± 3.7
PHB	69.1 ± 2.7	23.9 ± 2.6
PHB20	71.7 ± 2.9	42.5 ± 2.9
PHB30	72.0 ± 3.7	47.8 ± 2.8
PHBV	70.6 ± 5.3	23.0 ± 2.9
PHBV20	71.6 ± 1.5	34.0 ± 2.9
PHBV30	71.4 ± 3.5	26.8 ± 3.7
PHBV40	71.7 ± 1.8	47.2 ± 4.4

**Table 2:** The inhibition coefficient (Q) for the three variants of biocomposites.

Sample Code	Q
PLA30	1.84 ± 0.31
PHB20	1.23 ± 0.19
PHBV40	1.53 ± 0.21

## Data Availability

Data is contained within the article or Supplementary Material.

## Supplementary Materials

The following supporting information can be downloaded at: https://www.mdpi.com/article/10.3390/polym18202576/s1, Figure S1: TGA thermograms of BSG, neat PLA, PHB, PHBV and their biocomposites with BSG recorded under a nitrogen flow at heating rate of 20 °C/min.; Figure S2: Scanning electron micrographs of cryo-fracture surfaces of neat biopolymer carriers: PLA, PHB and PHBV.; Figure S3: Scanning electron micrographs of cryo-fracture surfaces of biocomposite carriers: PLA40, PHB30, and PHBV40.; Figure S4: DSC cooling thermograms of PLA-based carriers.; Figure S5: DSC cooling thermograms of PHB and PHBV-based carriers.; Table S1: Yeast multiplication on PLA, PHB and PHBV-based carriers after 6-days cultivation.
