# Supplementary material for: Biopolymer Composites Filled with Brewer’s Spent Grain for Yeast Immobilization
Source: Polymers (Basel). 2026 Oct 19;18(20):2576. doi: 10.3390/polym18202576 (PMC13708820; doi:10.3390/polym18202576)
Supplement: Supplementary file 1 [file polymers-18-02576-s001.zip › polymers-4583697-supplementary.pdf]

Supplementary Materials

Biopolymer Composites Filled with Brewer's Spent Grain for Yeast Immobilization

Jiayue Liu[1,2], Witold Fortuniak[3] , Joanna Bojda[3], Joanna Zakrzewska[3], Anna Rygała[2], Ewa Piorkowska[3*], Dorota Kręgiel[2], Urszula Mizerska[3*]

[1] Interdisciplinary Doctoral School; Lodz University of Technology, Żeromskiego 116, 90-924 Lodz, Poland; jiayue.liu@dokt.p.lodz.pl

[2] Department of Environmental Biotechnology, Faculty of Biotechnology and Food Sciences, Lodz University of Technology, Wolczanska 171/173, 90-924 Lodz, Poland.

[3] Centre of Molecular and Macromolecular Studies Polish Academy of Sciences, Sienkiewicza 112, 90-363 Lodz, Poland. urszula.mizerska@cbmm.lodz.pl*, ewa.piorkowska@cbmm.lodz.pl*

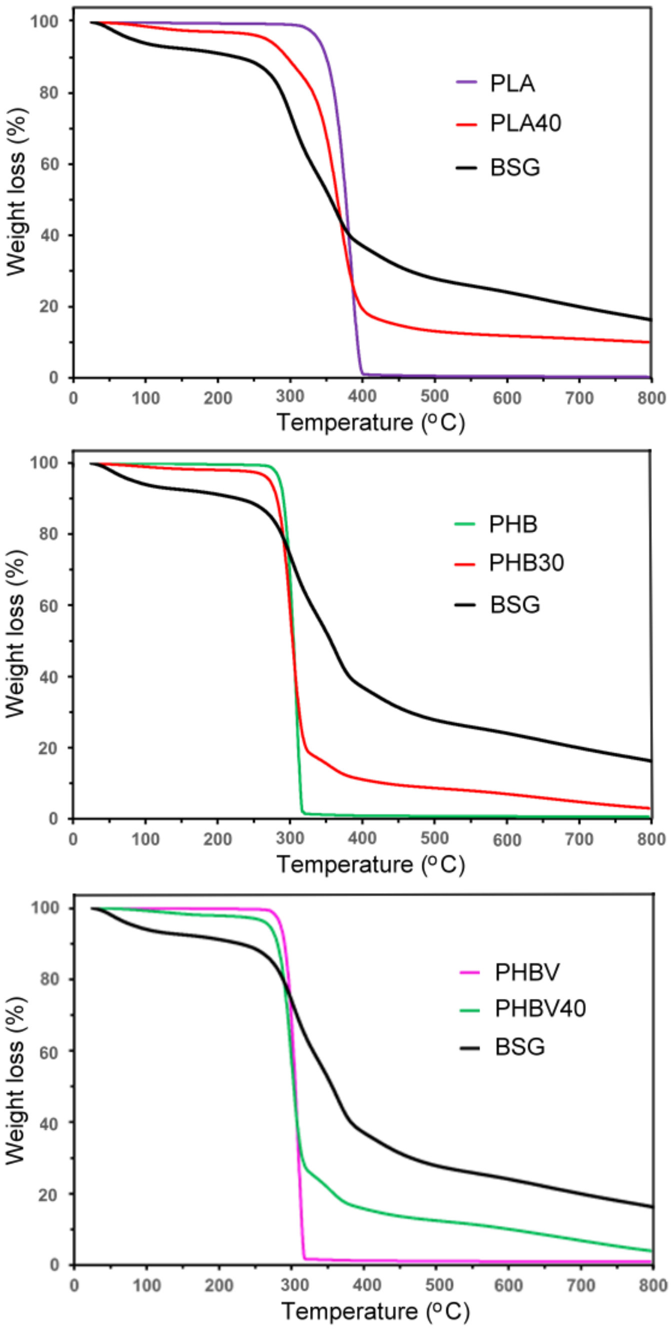

Figure S1. TGA thermograms of BSG, neat PLA, PHB, PHBV and their biocomposites with BSG recorded under a nitrogen flow at heating rate of 20 °C/min.

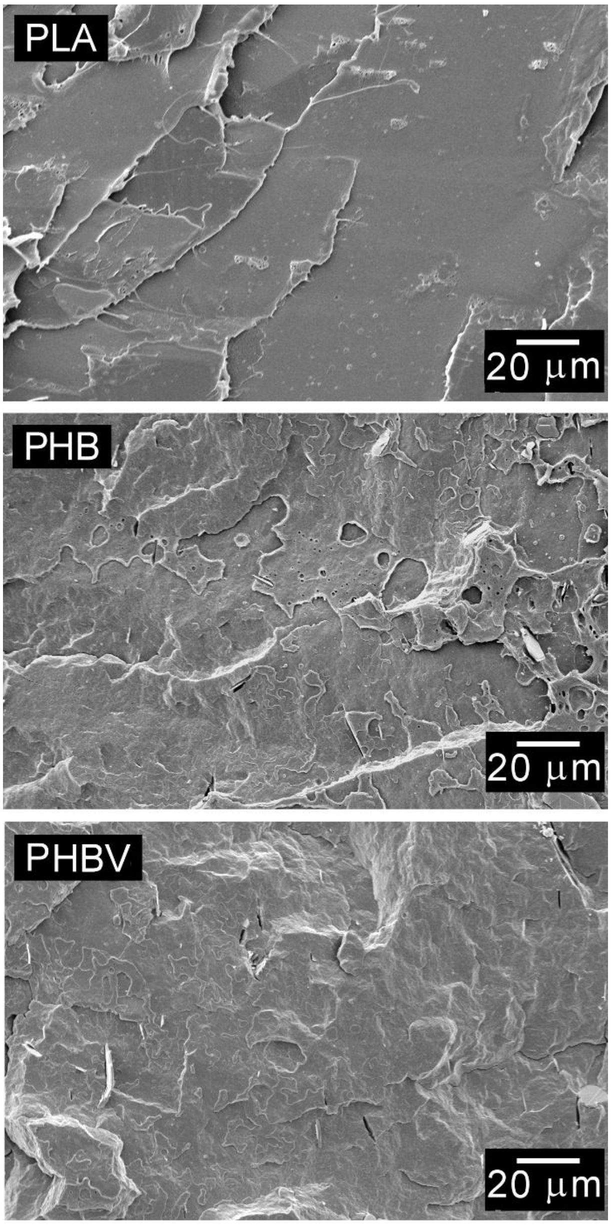

Figure S2. Scanning electron micrographs of cryo-fracture surfaces of neat biopolymer carriers: PLA, PHB and PHBV.

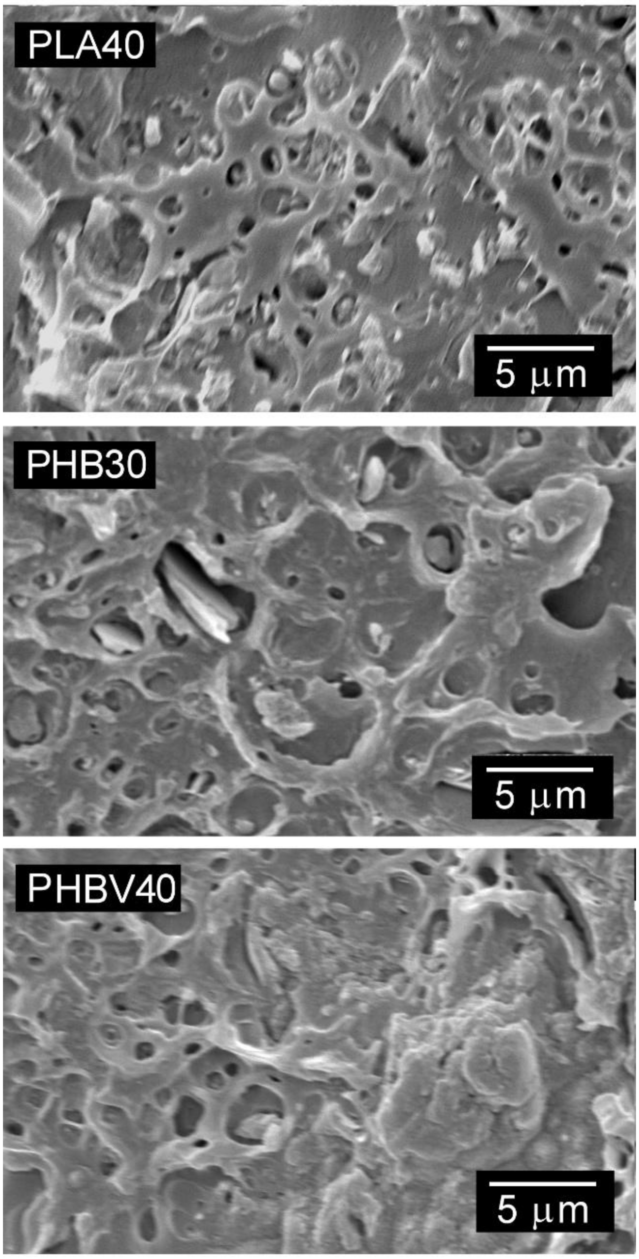

Figure S3. Scanning electron micrographs of cryo-fracture surfaces of biocomposite carriers: PLA40, PHB30, and PHBV40.

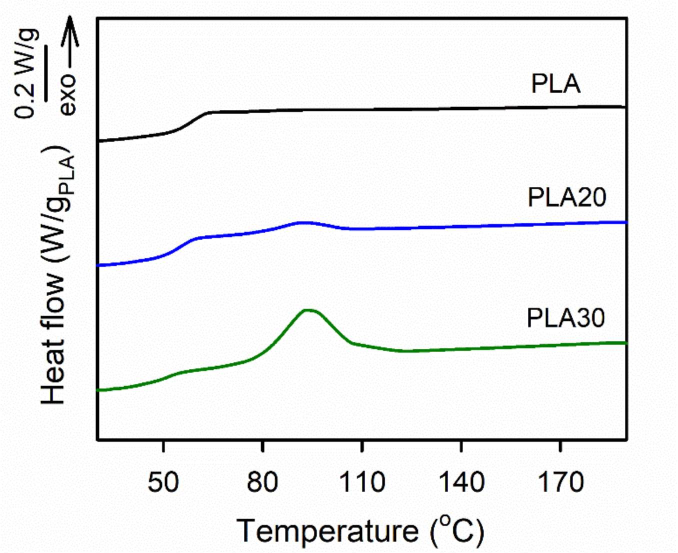

Figure S4. DSC cooling thermograms of PLA-based carriers.

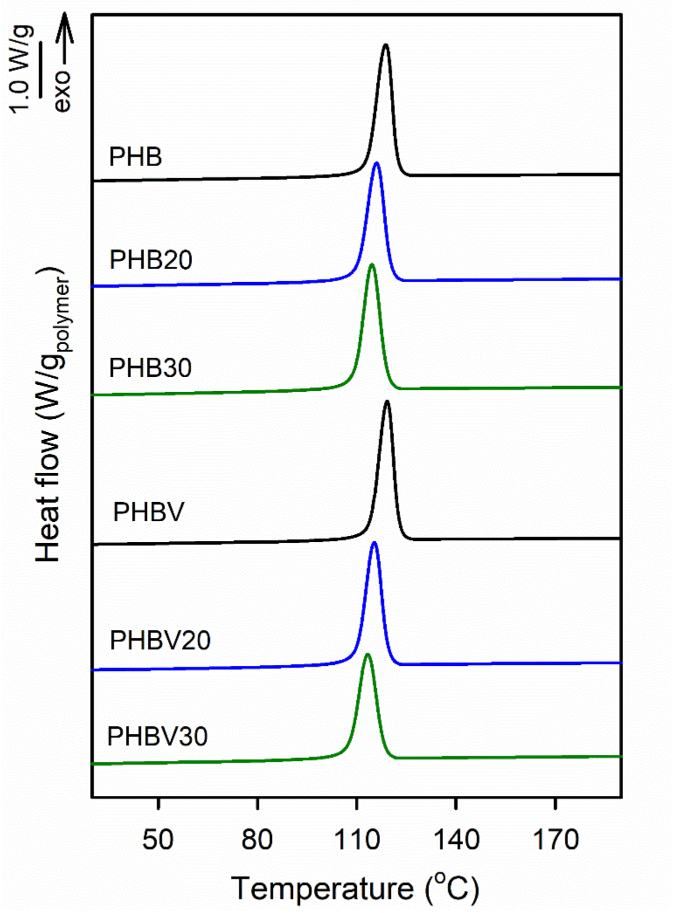

Figure S5. DSC cooling thermograms of PHB and PHBV-based carriers.

Table S1. Yeast multiplication on PLA, PHB and PHBV-based carriers after 6-days cultivation.

Sample code	Number of cells
PLA	2.29×10^7
PLA20	7.59×10^7
PLA30	9.33×10^7
PLA40	7.08×10^7
PHB	2.95×10^7
PHB20	2.45×10^7
PHB30	1.51×10^7
PHBV	1.62×10^7
PHBV20	2.63×10^7
PHBV30	2.63×10^7
PHBV40	4.26×10^7
